# Traumatic tibia shaft fractures caused by the impact of a golf ball: two case reports 

**DOI:** 10.1186/s13256-018-1771-3

**Published:** 2018-08-25

**Authors:** Yong-Chan Ha, Yu-Jun Kwon, Jun-Il Yoo, Ji-Seok Kim

**Affiliations:** 1 Department of Orthopaedic Surgery, Chung-Ang University College of Medicine, Seoul, South Korea; 2 Department of Orthopedic Surgery, U & J Hospital, KimPo, Republic of Korea; 3 Department of Orthopaedic Surgery, School of Medicine, Gyeongsang National University Hospital, Gyeongsang National University, 90 Chilamdong, Jinju, Gyeongnamdo 660-702 South Korea; 4 Department of Physical Education, Gyeongsang National University, Jinju, South Korea

**Keywords:** Fracture, Golf ball, Impacting power, Injury, Tibia shaft

## Abstract

**Background:**

As golf becomes increasingly popular, the number of injuries while playing golf also increases. We experienced two cases of traumatic tibia shaft fractures caused by the impact of a golf ball.

**Case presentation:**

A 48-year-old Korean man and a 43-year-old Korean man were diagnosed as having linear tibia shaft fractures on the right leg and left leg, respectively. Both of them were treated with closed intramedullary nails. Calculated impact power of the golf ball at the time of tibia fracture was 3372 and 5825 N, respectively. Radiologic and clinical complications such as nonunion and delayed union were not found up to the latest follow-up at 12 months postoperatively.

**Conclusions:**

Players and watchers of golf must take precautionary measures before striking a golf ball. It is advisable that players watching a golfer hitting a golf ball should stand a long way behind or in front of the golfer striking the golf ball. The danger of such injury is on the rise because more and more people are enjoying golf nowadays. Warning players of such dangers is one preventive measure to avoid such injuries in the future.

## Background

Golf is a popular recreational sport worldwide. According to estimates, in early 2008 there were at least 58 million regular golfers throughout the world, including 3 million golfers in Korea. Participation in golf has no gender or age limit [1, 2]; however, a decline in physical skills and abilities is inevitable with increasing age. As golf skills require muscle endurance, strength, flexibility, and cardiovascular fitness to some extent, all golfers are susceptible to possible injuries. Because golf is a relatively low-intensity sport with low physical demands, it is not associated with a very high risk of injury. However, a large number of golfers are older people in poor physical condition. Therefore, the number of golf-related injuries could still be high [3–5].

The most common self-reported mechanisms of injury are overuse followed by technical error. Other relatively common mechanisms include contact with a static object and a sudden or rapid change of club speed. However, traumatic tibia shaft fractures caused by the impact of a golf ball have not been reported in the literature.

Here, we report two cases of tibia shaft fracture caused by the impact of golf ball while playing golf. The impact power of a golf ball and the injury mechanism are also analyzed.

## Case presentation

### Case 1

**Fig. 1:**
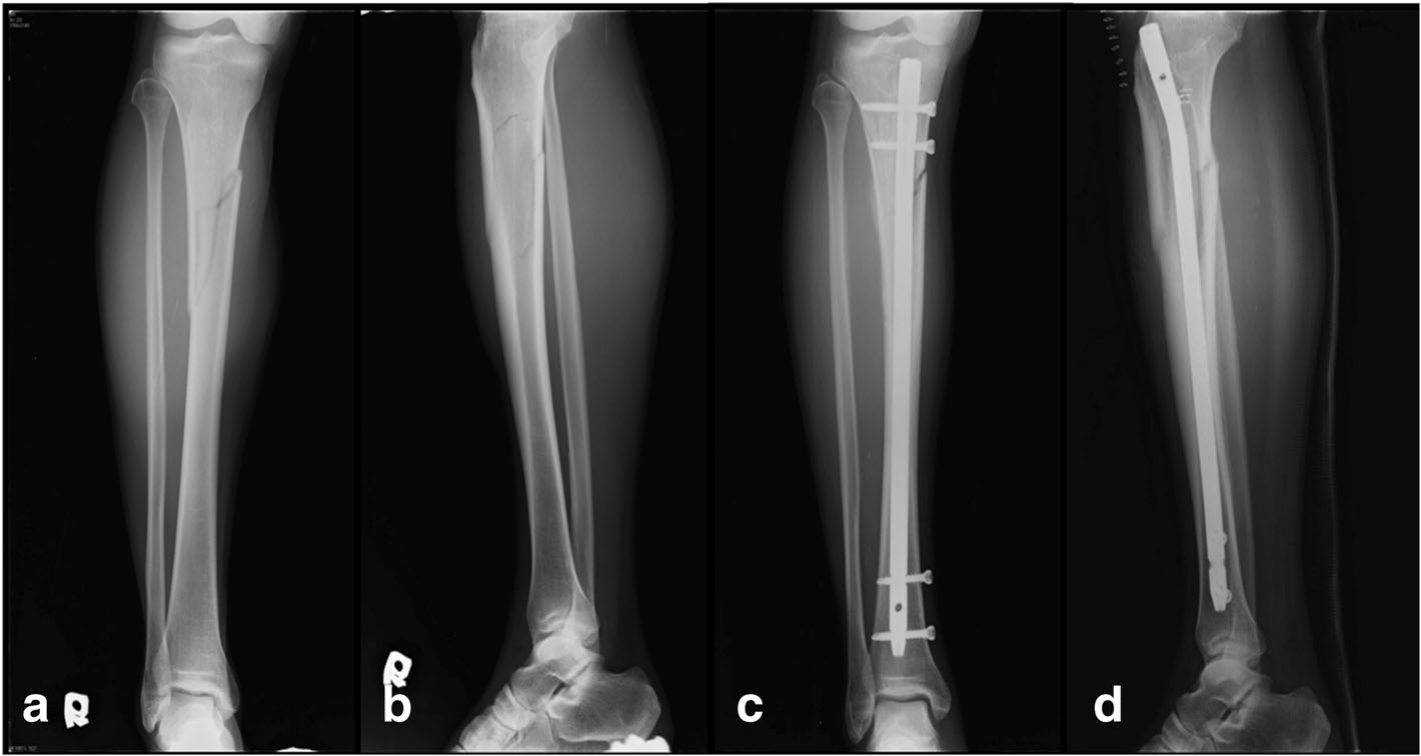
**a**, **b** A 48-year-old man with fracture in the lower third of the tibia shaft. **c**, **d** Closed reduction and intramedullary nailing was carried out after the injury

The patient was a 48-year-old Korean man among four players who were enjoying a golf game. On the 11th hole, one of the players swung a number 5 wood club to take his second shot. At the time, our patient was watching the shot approximately 10 meters away from the player at a 50 degree angle. The player was an experienced golfer who had played golf as a professional for over 10 years. Our patient fell down after being hit by a high speed golf ball on his lower leg. He presented to our hospital with severe pain in his lower extremity. There was no medical, family, and psychosocial history. An X-ray examination revealed a displaced fracture of the proximal one-third of the tibia (Fig. 1 a, b). He was treated with an intramedullary nail (Fig. 1c, d). He had postoperative follow-up at 6 weeks, 3 months, 6 months, 9 months, 12 months, and then yearly.

### Case 2

**Fig. 2:**
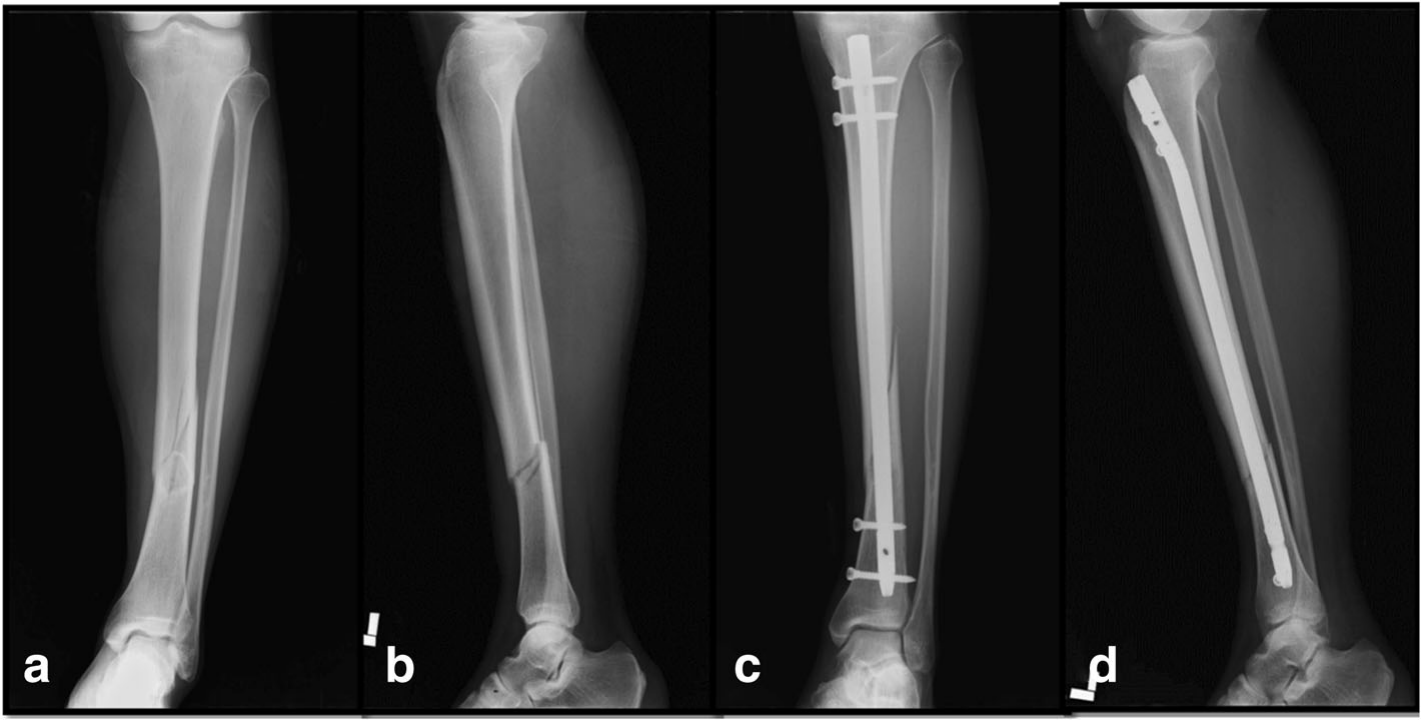
**a**, **b** Anteroposterior and lateral radiographs of a tibia fracture in a 43-year-old man. Plain films showing a fracture at the junction of the proximal and one third of the tibia. **c**, **d** Bone union following intramedullary nailing shown in anteroposterior and lateral views

A 43-year-old Korean man was one of four players who were enjoying a golf game. On the 12th hole, one of the players took a second shot with a wood club. The one who was making the swing was an inexperienced golfer. Our patient was watching the shot around 5 meters away, 15 degrees left of the player. The golf ball hit by the inexperienced player hit the lower leg of our patient. He was transferred to our hospital. He complained of severe pain of lower extremity. There was no medical, family, and psychosocial history. An X-ray examination revealed that he had a displaced fracture of the distal one-third of the tibia (Fig. 2a, b). He was treated with an intramedullary nail (Fig. 2c, d). He had postoperative follow-up at 6 weeks, 3 months, 6 months, 9 months, 12 months, and then yearly.

## Discussion and conclusions

One of the most common mechanisms of golf injuries is overuse. The most frequent nature of injury reported is strains. The parts of the body that are most often injured are the lower back and the shoulder [4, 6, 7]. There have been many reports of golf injuries; however, tibia fracture due to being hit by a golf ball has not been reported yet.

If the details of the golfing actions that caused the injuries to Case 1 and Case 2 are substituted into a formula for average power and taking into account drag force, then the force of the golf ball on impact was found to be approximately 3372 and 5825 N, respectively. In the two cases reported here, both patients were standing at a 15–50 degree angle from the player. Both players were taking their second shot with a wooden club. A ball hit by a wooden club can gain extreme speed, creating a lot of energy. An amateur golfer’s average club head speed is 90 miles per hour (40 m/s). Assuming that the weight of a general wooden club is 200 g and a golf ball’s weight is 45 g, at a shooting angle of 5 degrees the speed of the ball after the impact is calculated to be 16.3–65.3 m/s. The movement of a golf ball can have two physical conditions: inelastic collision and motion by drag force [8, 9].

### Inelastic collision

Conditions

$$ {\nu}_c^i: inital\ velocity\ of\ a\  golf\ club $$

$$ {\nu}_c^f: final\ velocity\ of\ a\  golf\ club $$

$$ {\nu}_b^i: inital\ velocity\ of\ a\  golf\ ball $$

$$ {\nu}_b^f: final\ velocity\ of\ a\  golf\ ball $$

$$ {\nu}_h^i: inital\ velocity\ of\ a\  human $$

$$ {\nu}_h^f: final\ velocity\ of\ a\  human $$

The masses of the three particles (golf club, golf ball, and human) are *m*_c_ = 0.2 kg, *m*_b_ = 0.045 kg, and *m*_h_ ≈ 60 kg.

1 $$ {m}_c{v}_c^i+{m}_b{v}_b^i={m}_c{v}_c^f+{m}_b{v}_b^f $$

2 $$ e=-\frac{v_c^f-{v}_b^f}{v_c^i-{v}_b^i} $$

The example gives a restitution coefficient of ball: e = 0.85. Put the coefficient into Eq. (2) and solve the simultaneous equation.

3 $$ {\displaystyle \begin{array}{l}{v}_b^f=\frac{m_c{v}_c^i\left(1+e\right)}{m_c+{m}_b}\\ {}\kern2.5em =\frac{0.2\times 40\left(1+0.01\right)}{0.2+0.045}\\ {}\kern2.5em \approx 60.40\left(m/{s}^2\right)\end{array}} $$

The ball moves at a constant velocity for the *x*-axis. The time for flight of the ball is:

4 $$ {t}_1=\frac{5m}{v_b^f\cos \theta }=0.082s $$

5 $$ {t}_2=\frac{10m}{v_b^f\cos \theta }=0.165s $$

For the above time, some of the air resistance is negligible. We can use the final velocity of the first collision ${\nu}_{b2}^i={\nu}_b^f\cos \theta .$ Therefore, the initial velocity of the golf ball in the second collision becomes

6 $$ {\nu}_{b2}^f=\frac{\left({m}_b-{e}^{\prime }{m}_h\right){\nu}_{b2}^i}{m_b+{m}_h} $$

7 =−18.06.

Assuming that the mass of a human is 60 kg, the inelastic collision coefficient *e'* is 0.3, and cos*θ* ≈ 1.

*e'* can be induced from the following definition;

8 $$ {e}^{\prime }=\frac{\left|\Delta  {v}_f\right|}{\left|\Delta  {v}_i\right|}=\sqrt{\frac{h_f}{h_i}}. $$

If we drop a golf ball at the height of h reference to the ground, the distance of the ball after collision is quite short. Therefore, the collision coefficient of the second collision is less than that for the first one. The difference of the momentum ∆*p*_*ball*_ equals the impulse,

9 $$ \Delta  {p}_{ball}=0.045\times \left\{60.40-\left(-18.06\right)\right\}=78.47 $$

10 $$ \Delta  {p}_{ball}={F}_{avg}\times \Delta  t $$

We cannot calculate the collision time because an experiment is required for such a measurement. If we assume that the collision time is the same as that in the first collision, ∆t = 0.5ms.

11 $$ {F}_{avg}=\frac{3.53}{0.5\times {10}^{-3}}=7062N $$

### Consider the drag force

If we consider the air resistance, there is a drag force (D=$\frac{1}{2} C\rho A{v}^2$) against the movement of a particle.

12 $$ \mathrm{F}=\mathrm{ma}=-\mathrm{D} $$

13 $$ \kern3em \mathrm{ma}=-\frac{1}{2} C\rho A{v}^2 $$

14 $$ \kern2.25em \frac{1}{v^2}\frac{dv}{dt}=-\frac{b}{m},\left(b=\frac{1}{2} C\rho A\right) $$

15 $$ \kern0.75em {\int}_0^{t^{\ast }}\frac{1}{v^2}\frac{dv}{dt}={\int}_0^{t^{\ast }}-\frac{b}{m} dt $$

16 $$ {\int}_{v_0}^{v_{t^{\ast }}}\frac{1}{v^2} dv=-\frac{b}{m}{t}^{\ast } $$

17 $$ \kern1.5em \therefore \left({t}^{\ast}\right)={\left[\frac{2b}{m}{t}^{\ast }+\frac{1}{v_0}\right]}^{-1} $$

where *v*_0_ is the initial velocity of a particle, t* is the time of flight (t_1_ = 0.082 s and t_2_ = 0.165 s). $\mathrm{D}=\frac{1}{2} C\rho A{v}^2$ is the drag force, where *C* is the drag coefficient, *ρ* is density of air, and *A* is an effective area. We can use Eq. (17) to calculate ${\nu}_b^f$just before the second collision. Usually, the drag coefficient *C* is determined by experiment (0.4 ~ 0.1). In this case, however, the radius of a golf ball is quite small and the dimples affect the decrement of its drag coefficient. The drag force is calculated with radii_*ball*_ = 4.265 × 10^−2^ and density of air *ρ* = 1.293kg/*m*^3^. ${\nu}_b^f$, the final velocity after first collision, was calculated under the coefficient of restitution, *ℯ* = 0.1 and *F*_*avg*_ takes account of the collision time ∆*t* = 5ms. If the ball is a perfect sphere the coefficient becomes *C* = 0.47 (Table 1).

**Table 1:** Variables of calculating final velocity of a golf ball

C	0.47	0.235	0.1	0.01	0
$\frac{1}{2} C\rho Av$	1.61 × 10^−3^	8.06 × 10^−4^	3.43 × 10^−4^	3.43 × 10^−5^	0.00
${\nu}_b^f\ \left(5\mathrm{m}\right)$ ${\nu}_b^f\ \left(10\mathrm{m}\right)$	44.58 34.09	50.63 43.58	55.82 51.88	59.91 59.42	60.40 60.40
*F*_*avg*_ (5*m*) *F*_*avg*_ (10*m*)	5825 3372	5888 4312	5935 5133	5971 5879	5975 5975

*C* drag coefficient, *F*_*avg*_ impulsive force, *ρ* density of air,${\nu}_b^f$ final velocity

In this case, the impact power of the golf ball as it hit the patients is calculated as 3372 and 5825 N, respectively.

In conclusion, both players and watchers must take precautionary measures before striking the golf ball. It is advisable that the players watching the golfer striking the golf ball should stand a long way behind or in front of the golfer hitting the golf ball. The danger of such an injury is on the rise because more and more people are enjoying golf nowadays. Warning players of such dangers is one preventive measure to avoid such injuries in the future.
